# Differential effects of the blood pressure state on pulse rate variability and heart rate variability in critically ill patients

**DOI:** 10.1038/s41746-021-00447-y

**Published:** 2021-05-14

**Authors:** Elisa Mejía-Mejía, James M. May, Mohamed Elgendi, Panayiotis A. Kyriacou

**Affiliations:** 1 Research Centre for Biomedical Engineering, City, University of London, London, United Kingdom; 2 Rady Faculty of Health Sciences, University of Manitoba, Winnipeg, MB Canada

**Keywords:** Autonomic nervous system, Biomedical engineering

## Abstract

Heart rate variability (HRV) utilizes the electrocardiogram (ECG) and has been widely studied as a non-invasive indicator of cardiac autonomic activity. Pulse rate variability (PRV) utilizes photoplethysmography (PPG) and recently has been used as a surrogate for HRV. Several studies have found that PRV is not entirely valid as an estimation of HRV and that several physiological factors, including the pulse transit time (PTT) and blood pressure (BP) changes, may affect PRV differently than HRV. This study aimed to assess the relationship between PRV and HRV under different BP states: hypotension, normotension, and hypertension. Using the MIMIC III database, 5 min segments of PPG and ECG signals were used to extract PRV and HRV, respectively. Several time-domain, frequency-domain, and nonlinear indices were obtained from these signals. Bland–Altman analysis, correlation analysis, and Friedman rank sum tests were used to compare HRV and PRV in each state, and PRV and HRV indices were compared among BP states using Kruskal–Wallis tests. The findings indicated that there were differences between PRV and HRV, especially in short-term and nonlinear indices, and although PRV and HRV were altered in a similar manner when there was a change in BP, PRV seemed to be more sensitive to these changes.

## Introduction

Heart rate variability (HRV), which is defined as changes in heart rate over time^1^, is an indicator that is used to evaluate the activity of the cardiac autonomic nervous system (ANS) because of its relationship with the parasympathetic and sympathetic activity directed into the sinus node in the heart, which controls the heart rate^2,3^. Several authors have found that HRV is associated with cardiovascular conditions, such as myocardial infarction and heart failure^4^, coronary artery disease, and sudden death^5^. As explained in^6^, HRV has also been found to aid in the diagnosis and increase the prognostic value of predisposing conditions for critical illness, such as hypertension, and some HRV parameters have been found to be abnormal even in the early stages of hypertension.

HRV is measured using electrocardiographic signals (ECG), which represent the electrical activity generated by the heart conduction system^3,7^, and standards of measurement have been established to align the methodologies used in HRV studies, in order to allow for comparisons among results^8^. Nonetheless, in recent years, several studies have reported obtaining information similar to HRV from other signals that also contain information related to the cardiac cycle, such as pulse waves. One technique that has attracted significant attention for detecting pulse-wave-related HRV, also known as pulse rate variability (PRV), is photoplethysmography (PPG)^9^, which is a noninvasive, simple, and inexpensive technique that utilizes optical principles to obtain the pulse wave from the microcirculation in peripheral tissue^10,11^. Various studies have investigated PRV changes under different conditions, such as in the presence of mental or somatic diseases or during sleep, and used them to evaluate the effects of pharmacological drugs on ANS responses^8^. There has also been a special interest in the study of PRV under cardiovascular conditions, such as diabetes, hypo- or hypertension, or cardiac arrhythmias^1^.

Although some studies have shown that PRV is a promising technique for identifying several conditions and that PRV is highly correlated with HRV, these results have mainly been observed in healthy or resting subjects in the supine position^1^. On the other hand, some studies have argued that PRV is not necessarily a good surrogate for HRV, mainly because of the errors made when performing the processing and acquisition methods and physiological factors, such as changes in pulse transit time (PTT)^1,12^. As explained in refs. ^13^ and ^14^, PTT plays an important role in the differences that are seen between PRV and HRV.

PTT is the time it takes for the pulse wave to travel from the heart to the peripheral tissue where it is being measured, and it has been shown to be related to blood pressure (BP)^15^. BP refers to the force that the heart uses to pump blood through the circulatory system and is one of the main measurements used to understand the behavior of the cardiovascular system^16^. Its associated abnormalities, especially hypertension (i.e., high BP), are associated with fatal cardiovascular diseases^17^.

Because of the relationship between PTT and BP and the effects of PTT on PRV measurements^13,14^, the aim of this study was to evaluate the relationship between HRV and PRV measured from ECG and PPG signals, respectively, obtained from critically ill patients with hypotension, hypertension, or normotension. It was hypothesized that HRV and PRV would not exhibit the same behavior and that their relationship would be affected by the BP state. To evaluate these hypotheses, signals obtained from the public MIMIC-III database from Physionet were analyzed, and PRV and HRV indices were extracted and compared to assess the relationship between HRV and PRV.

## Results

### Signal selection and segmentation

Data used in this study was obtained from the freely available MIMIC-III Waveform^18,19^. The dataset was filtered according to the length of the recordings and the quality of the ABP signals for each patient. In total, 230 records with poor-quality (PQ) ABP signals and a short duration were discarded. The signals from the remaining 270 records were used in the subsequent analysis. All signals were segmented in 5-min length segments with 10 s overlap between consecutive segments. In total, 4937 5-min segments were extracted, of which 54% were labeled as hypertensive, 25% were labeled as hypotensive, and the remaining 22% were labeled as normotensive events, according to the ABP signals.

### Extraction of indices

Time- and frequency-domain and nonlinear indices were obtained from HRV and PRV signals (Table 1). Table 2 summarizes the behavior of these indices when measured during hypotension, normotension, and hypertension.

**Table 1:** Indices extracted from pulse rate variability (PRV) and heart rate variability (HRV).

Indices		Description, units of measurement
Time-domain^7^	AVNN	Average value of the normal-to-normal beats (NN), s
	SDNN	Standard deviation of the NN, s
	RMSSD	Root mean squared value of successive differences of NN, s
	NN50	Number of interval differences of successive NN intervals greater than 50 ms
	pNN50	Proportion of NN50 divided by the total number of NN intervals
Frequency-domain^7^	VLF	Power of the power spectrum of interpolated PRV/HRV in the band between 0.0033 and 0.04 Hz, s^2^
	LF	Power of the power spectrum of interpolated PRV/HRV in the band between 0.04 and 0.15 Hz, s^2^
	HF	Power of the power spectrum of interpolated PRV/HRV in the band between 0.15 and 0.40 Hz, s^2^
	TP	Power of the power spectrum of interpolated PRV/HRV in the band between 0.0033 and 0.40 Hz, s^2^
	nLF	Normalized power of the LF band
	nHF	Normalized power of the HF band
	LF/HF	Ratio of the LF and HF bands
	cLF_x_	X coordinate of the centroid of the LF band, Hz
	cHF_x_	X coordinate of the centroid of the HF band, Hz
	cTP_x_	X coordinate of the centroid of the TP band, Hz
	cLF_y_	Y coordinate of the centroid of the LF band, s^2^
	cHF_y_	Y coordinate of the centroid of the HF band, s^2^
	cTP_y_	Y coordinate of the centroid of the TF band, s^2^
	SpEn	Spectral entropy of the power spectrum
Poincaré plot	S	Area of the ellipse formed in the Poincaré plot^43^, s^2^
	SD1	Dispersion of the Poincaré plot points perpendicular to the line of identity^43^, s
	SD2	Dispersion of the Poincaré plot points along the line of identity^43^, s
	SD1/SD2	Ratio between SD1 and SD2^43^
	COM	Normalized summation of the distances between each point of the Poincaré plot and its centroid
Entropy analysis	BSE	Basic scale entropy of PRV/HRV trends^44^
	SSE	Sign-series entropy of PRV/HRV trends^45,46^
	ApEn	Approximate entropy of PRV/HRV trends^47^
	SampEn	Sample entropy of PRV/HRV trends^47^
	MSE	Multiscale entropy of PRV/HRV trends^47^
Phase analysis^47^	D2	Correlation dimension of PRV/HRV trends
	LYA	Lyapunov exponent of PRV/HRV trends
DFA ^a48^	A1	Short-range scaling exponent, *α*_1_
	A2	Long-range scaling exponent, *α*_2_

^a^ Detrended fluctuation analysis.

**Table 2:** Mean ± standard deviation of indices measured from pulse rate variability (PRV) and heart rate variability (HRV) under each blood pressure state.

Indices	Hypotension		Normotension		Hypertension	
	PRV	HRV	PRV	HRV	PRV	HRV
AVNN ^a^	826.4 ± 104.5	826.0 ± 104.5	755.7 ± 136.6	755.4 ± 136.5	836.9 ± 93.9	836.5 ± 93.7
SDNN ^a^	10.8 ± 7.12	9.49 ± 7.12	14.4 ± 10.6	12.9 ± 10.5	16.3 ± 12.5	15.0 ± 12.7
RMSSD ^a^	11.8 ± 5.48	8.66 ± 4.33	14.7 ± 8.12	10.5 ± 6.23	15.6 ± 8.84	11.9 ± 7.64
NN50	2.58 ± 9.18	1.37 ± 6.97	4.88 ± 12.7	2.03 ± 9.21	6.13 ± 13.5	2.93 ± 8.03
pNN50 ^a^	6.25 ± 21.0	3.00 ± 14.1	12.7 ± 34.0	5.27 ± 24.9	16.9 ± 38.6	8.21 ± 23.2
VLF ^b^	1.31 ± 3.11	1.30 ± 3.20	1.54 ± 3.10	1.60 ± 3.18	4.21 ± 9.14	4.29 ± 9.44
LF ^b^	0.75 ± 1.30	0.68 ± 1.15	1.05 ± 2.10	0.98 ± 2.01	2.06 ± 4.13	1.97 ± 4.31
HF ^b^	1.44 ± 3.09	1.09 ± 2.75	1.69 ± 2.89	1.23 ± 3.06	2.15 ± 3.99	1.69 ± 3.93
TP ^b^	3.50 ± 5.81	3.07 ± 5.31	4.28 ± 7.43	3.82 ± 7.46	8.41 ± 15.1	7.95 ± 15.4
nLF	0.71 ± 0.39	0.95 ± 0.51	0.93 ± 0.82	1.55 ± 1.36	0.97 ± 0.74	1.26 ± 0.92
nHF	0.26 ± 0.10	0.28 ± 0.11	0.24 ± 0.10	0.26 ± 0.11	0.27 ± 0.11	0.28 ± 0.11
LF/HF	0.43 ± 0.17	0.36 ± 0.17	0.42 ± 0.22	0.34 ± 0.24	0.36 ± 0.17	0.30 ± 0.16
cLF_x_	0.09 ± 0.01	0.09 ± 0.01	0.08 ± 0.01	0.08 ± 0.01	0.09 ± 0.01	0.08 ± 0.01
cHF_x_	0.27 ± 0.01	0.27 ± 0.01	0.28 ± 0.02	0.27 ± 0.02	0.28 ± 0.02	0.27 ± 0.02
cTP_x_	0.15 ± 0.05	0.13 ± 0.05	0.14 ± 0.05	0.12 ± 0.06	0.13 ± 0.05	0.11 ± 0.05
cLF_y_^c^	6.84 ± 11.8	6.26 ± 9.84	10.9 ± 22.3	10.4 ± 21.5	24.0 ± 69.5	24.6 ± 81.1
cHF_y_^c^	8.15 ± 22.9	6.78 ± 23.0	15.4 ± 24.8	10.3 ± 19.5	9.23 ± 16.3	7.39 ± 16.6
cTP_y_^c^	48.3 ± 201.7	50.5 ± 204.4	35.1 ± 61.7	36.1 ± 66.1	105.6 ± 273.1	110.2 ± 279.0
SpEn	24.5 ± 1.65	24.2 ± 1.95	23.9 ± 1.29	23.2 ± 1.60	23.8 ± 1.91	23.4 ± 2.14
S	0.06 ± 0.03	0.05 ± 0.02	0.07 ± 0.05	0.05 ± 0.04	0.08 ± 0.05	0.06 ± 0.04
SD1 ^a^	8.32 ± 3.87	6.12 ± 3.06	10.4 ± 5.74	7.43 ± 4.41	11.1 ± 6.25	8.39 ± 5.40
SD2	2.34 ± 0.30	2.34 ± 0.30	2.14 ± 0.39	2.14 ± 0.30	2.37 ± 0.27	2.37 ± 0.27
SD1/SD2 ^a^	3.65 ± 2.00	2.70 ± 1.71	4.86 ± 2.51	3.48 ± 1.90	4.73 ± 2.71	3.59 ± 2.31
COM ^a^	0.34 ± 0.60	0.28 ± 0.58	0.64 ± 1.21	0.55 ± 1.14	0.84 ± 1.61	0.76 ± 1.59
BSE	5.65 ± 0.29	5.29 ± 0.45	5.68 ± 0.23	5.31 ± 0.40	5.71 ± 0.23	5.45 ± 0.36
SSE	2.69 ± 0.17	2.78 ± 0.12	2.66 ± 0.21	2.80 ± 0.14	2.63 ± 0.18	2.75 ± 0.15
ApEn	0.43 ± 0.13	0.40 ± 0.17	0.42 ± 0.12	0.36 ± 0.16	0.42 ± 0.13	0.38 ± 0.16
SampEn	0.45 ± 0.20	0.43 ± 0.25	0.40 ± 0.16	0.34 ± 0.19	0.42 ± 0.20	0.39 ± 0.23
MSE	4.99 ± 1.95	4.47 ± 2.16	4.63 ± 1.93	4.20 ± 1.88	4.86 ± 1.92	4.48 ± 1.98
D2	−0.54 ± 33.2	−0.98 ± 19.6	−1.12 ± 31.7	−0.34 ± 8.56	−1.19 ± 33.2	−1.31 ± 20.1
LYA	3.27 ± 1.10	3.03 ± 1.04	3.52 ± 1.18	3.13 ± 1.08	3.54 ± 1.12	3.34 ± 1.14
A1	0.67 ± 0.39	0.77 ± 0.65	0.68 ± 0.18	0.80 ± 0.25	0.74 ± 0.21	0.84 ± 0.25
A2	0.87 ± 0.25	0.91 ± 0.27	0.90 ± 0.24	0.94 ± 0.23	0.88 ± 0.23	0.91 ± 0.24

^a^ Values multiplied by 1 × 10^−3^.

^b^ Values divided by 1 × 10^9^.

^c^ Values divided by 1 × 10^6^.

**Table 3:** Friedman rank sum tests results for the comparison between pulse rate variability and heart rate variability in the different blood pressure states. ‡: *p* value less than 5.00 × 10^−2^; †: *p* value less than 5.00 × 10^−3^; ⋆: *p* value less than 5.00 × 10^−4^.

Indices	Friedman rank sum test *p* values		
	Hypotension	Normotension	Hypertension
AVNN	9.48 × 10^−14^⋆	6.24 × 10^−10^⋆	1.53 × 10^−21^⋆
SDNN	3.32 × 10^−72^⋆	2.01 × 10^−50^⋆	9.98 × 10^−169^⋆
RMSSD	1.21 × 10^−181^⋆	1.40 × 10^−158^⋆	0.00⋆
NN50	7.25 × 10^−22^⋆	2.30 × 10^−50^⋆	1.01 × 10^−134^⋆
pNN50	1.23 × 10^−22^⋆	5.56 × 10^−52^⋆	8.87 × 10^−137^⋆
VLF	2.48 × 10^−3^†	4.83 × 10^−1^	2.42 × 10^−9^⋆
LF	3.78 × 10^−34^⋆	3.27 × 10^−46^⋆	8.82 × 10^−89^⋆
HF	7.96 × 10^−180^⋆	4.73 × 10^−160^⋆	0.00⋆
TP	1.86 × 10^−79^⋆	7.94 × 10^−73^⋆	1.00 × 10^−159^⋆
nLF	4.39 × 10^−17^⋆	6.89 × 10^−19^⋆	5.16 × 10^−16^⋆
nHF	1.12 × 10^−136^⋆	1.07 × 10^−151^⋆	3.10 × 10^−236^⋆
LF/HF	3.62 × 10^−109^⋆	1.31 × 10^−129^⋆	4.12 × 10^−189^⋆
cLF_x_	3.05 × 10^−14^⋆	6.61 × 10^−24^⋆	2.49 × 10^−66^⋆
cHF_x_	4.05 × 10^−22^⋆	1.44 × 10^−30^⋆	6.27 × 10^−66^⋆
cTP_x_	5.12 × 10^−134^⋆	8.87 × 10^−163^⋆	4.34 × 10^−250^⋆
cLF_y_	1.17 × 10^−19^⋆	8.72 × 10^−24^⋆	3.47 × 10^−34^⋆
cHF_y_	1.13 × 10^−142^⋆	7.43 × 10^−136^⋆	1.33 × 10^−242^⋆
cTP_y_	1.40 × 10^−7^⋆	4.31 × 10^−1^	1.61 × 10^−9^⋆
SpEn	5.09 × 10^−56^⋆	1.32 × 10^−90^⋆	1.74 × 10^−172^⋆
S	4.08 × 10^−183^⋆	5.69 × 10^−161^⋆	0.00⋆
SD1	1.21 × 10^−181^⋆	1.40 × 10^−158^⋆	0.00⋆
SD2	8.76 × 10^−14^⋆	5.67 × 10^−10^⋆	1.42 × 10^−21^⋆
SD1/SD2	1.69 × 10^−179^⋆	3.20 × 10^−156^⋆	0.00⋆
COM	2.75 × 10^−73^⋆	2.70 × 10^−50^⋆	1.56 × 10^−169^⋆
BSE	1.35 × 10^−262^⋆	1.58 × 10^−230^⋆	0.00⋆
SSE	2.22 × 10^−77^⋆	7.27 × 10^−114^⋆	1.34 × 10^−251^⋆
ApEn	2.58 × 10^−21^⋆	8.74 × 10^−57^⋆	4.37 × 10^−83^⋆
SampEn	2.44 × 10^−19^⋆	8.46 × 10^−56^⋆	4.03 × 10^−88^⋆
MSE	3.63 × 10^−30^⋆	2.21 × 10^−29^⋆	9.07 × 10^−30^⋆
D2	1.09 × 10^−11^⋆	3.28 × 10^−22^⋆	1.00 × 10^−14^⋆
LYA	1.18 × 10^−12^⋆	7.83 × 10^−18^⋆	5.71 × 10^−16^⋆
A1	1.71 × 10^−102^⋆	1.04 × 10^−101^⋆	8.73 × 10^−215^⋆
A2	5.62 × 10^−25^⋆	2.89 × 10^−29^⋆	1.45 × 10^−34^⋆

### Correlation between PRV and HRV indices

**Fig. 1:**
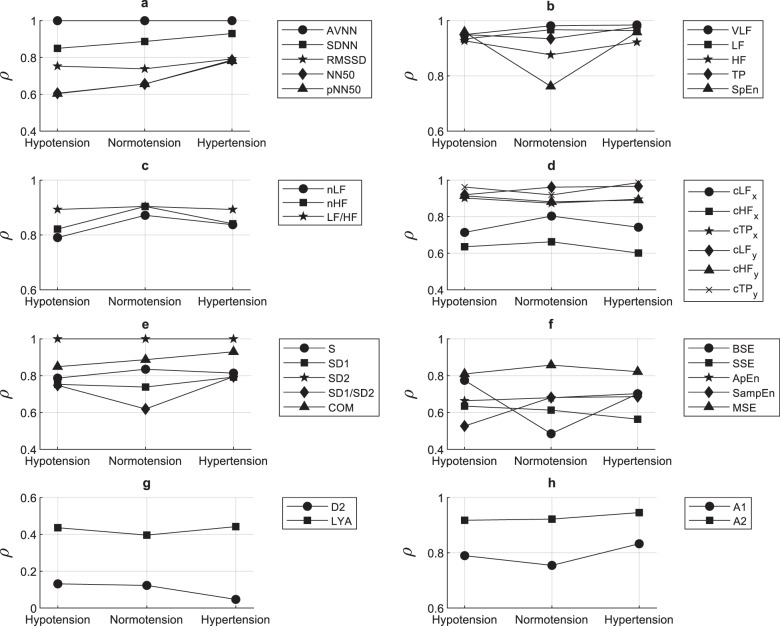
Linear relationship between pulse rate variability and heart rate variability. Spearman correlation coefficients (*ρ*) were measured between **a** time-domain indices, **b** absolute and entropy indices from the frequency domain, **c** relative indices from the frequency domain, **d** centroid-related indices from the frequency domain, **e** Poincaré plot indices, **f** entropy-related indices, **g** phase-related indices, and **h** indices resulting from the detrended fluctuation analysis. All indices were obtained from pulse rate variability and heart rate variability in each blood pressure state (hypotension, normotension, and hypertension).

The correlation between indices measured from PRV and HRV was assessed and the obtained results are summarized in Fig. 1. Most of the indices showed a good correlation between HRV and PRV during all three BP states, although some of the indices tended to show a lower correlation during normotension. Interestingly, the entropy- and phase-derived indices had lower correlations. In addition, lower correlation coefficients were observed for indices associated with short-term changes, such as RMSSD, SD1, HF, and A1.

### Comparison between HRV and PRV using the Friedman rank sum test

Because most of the data obtained from the different indices were non-normally distributed and did not comply with the assumption of homogeneity of variances, Friedman rank sum tests were used as a nonparametric alternative to repeated measures ANOVA to compare the indices measured from HRV and PRV under different BP states. The results from these tests for the comparison between HRV and PRV are shown in Table 3. For most indices, there were significant differences (*p* value < 0.001) between HRV and PRV, regardless of the BP state. Only VLF and cTPy showed nonsignificant differences between the two measurement sources during normotension.

**Table 4:** Kruskal–Wallis and post hoc multiple comparisons *p* values for the comparison among blood pressure states from indices measured from pulse rate variability. ‡: *p* value less than 5.00 × 10^−2^; †: *p* value less than 5.00 × 10^−3^; ⋆: *p* value less than 5.00 × 10^−4^.

Indices	KW ^a^	Multiple comparisons		
		PW1 ^b^	PW2 ^c^	PW3 ^d^
AVNN	2.1 × 10^−60^⋆	7.5 × 10^−36^⋆	6.3 × 10^−1^	3.8 × 10^−59^⋆
SDNN	9.8 × 10^−57^⋆	1.0 × 10^−25^⋆	1.8 × 10^−55^⋆	1.9 × 10^−4^⋆
RMSSD	1.1 × 10^−53^⋆	2.8 × 10^−20^⋆	1.4 × 10^−55^⋆	1.6 × 10^−3^†
NN50	8.5 × 10^−43^⋆	1.7 × 10^−6^⋆	6.6 × 10^−41^⋆	8.7 × 10^−12^⋆
pNN50	1.5 × 10^−43^⋆	1.3 × 10^−6^⋆	1.4 × 10^−41^⋆	5.6 × 10^−12^⋆
VLF	2.7 × 10^−70^⋆	1.5 × 10^−14^⋆	7.6 × 10^−64^⋆	3.5 × 10^−21^⋆
LF	5.1 × 10^−75^⋆	2.5 × 10^−4^⋆	1.1 × 10^−66^⋆	2.1 × 10^−30^⋆
HF	9.2 × 10^−32^⋆	1.6 × 10^−8^⋆	1.3 × 10^−33^⋆	2.8 × 10^−4^⋆
TP	1.3 × 10^−62^⋆	2.5 × 10^−11^⋆	1.6 × 10^−57^⋆	2.9 × 10^−19^⋆
nLF	4.7 × 10^−5^⋆	2.7 × 10^−4^⋆	7.4 × 10^−1^	2.3 × 10^−4^⋆
nHF	4.2 × 10^−29^⋆	6.1 × 10^−3^‡	1.9 × 10^−29^†	1.5 × 10^−8^†
LF/HF	4.8 × 10^−20^⋆	6.2 × 10^−1^	5.0 × 10^−19^⋆	2.8 × 10^−8^⋆
cLF_x_	8.8 × 10^−12^⋆	5.5 × 10^−12^⋆	2.1 × 10^−4^⋆	1.8 × 10^−5^⋆
cHF_x_	1.6 × 10^−7^⋆	3.5 × 10^−5^⋆	4.6 × 10^−7^⋆	6.9 × 10^−1^
cTP_x_	2.0 × 10^−21^⋆	1.2 × 10^−2^‡	3.1 × 10^−21^⋆	7.8 × 10^−7^⋆
cLF_y_	3.2 × 10^−73^⋆	1.3 × 10^−6^⋆	4.4 × 10^−67^⋆	3.1 × 10^−26^⋆
cHF_y_	1.3 × 10^−40^⋆	3.0 × 10^−29^⋆	3.8 × 10^−31^⋆	1.6 × 10^−6^⋆
cTP_y_	1.1 × 10^−56^⋆	2.5 × 10^−43^⋆	2.5 × 10^−48^⋆	2.3 × 10^−1^
SpEn	2.5 × 10^−40^⋆	4.2 × 10^−39^⋆	5.8 × 10^−28^⋆	8.3 × 10^−3^‡
S	1.3 × 10^−49^⋆	2.3 × 10^−6^⋆	8.6 × 10^−51^⋆	6.8 × 10^−12^⋆
SD1	1.1 × 10^−53^⋆	2.8 × 10^−20^⋆	1.4 × 10^−55^⋆	1.6 × 10^−3^†
SD2	2.0 × 10^−60^⋆	7.9 × 10^−36^⋆	6.2 × 10^−1^	3.6 × 10^−59^⋆
SD1/SD2	1.2 × 10^−64^⋆	4.9 × 10^−53^⋆	2.8 × 10^−51^⋆	2.3 × 10^−2^‡
COM	1.1 × 10^−56^⋆	8.8 × 10^−26^⋆	2.1 × 10^−55^⋆	2.0 × 10^−4^⋆
BSE	1.4 × 10^−7^⋆	9.5 × 10^−1^	2.5 × 10^−6^⋆	1.7 × 10^−4^⋆
SSE	7.1 × 10^−24^⋆	4.4 × 10^−2^‡	2.2 × 10^−23^⋆	3.0 × 10^−8^⋆
ApEn	1.1 × 10^−6^⋆	1.2 × 10^−5^⋆	9.5 × 10^−6^⋆	1.0
SampEn	8.2 × 10^−10^⋆	9.2 × 10^−11^⋆	1.4 × 10^−5^⋆	2.8 × 10^−2^‡
MSE	1.1 × 10^−5^⋆	6.0 × 10^−6^⋆	1.2 × 10^−2^‡	1.5 × 10^−2^‡
D2	5.7 × 10^−3^‡	4.0 × 10^−1^	3.5 × 10^−3^†	7.0 × 10^−1^
LYA	2.9 × 10^−14^⋆	6.9 × 10^−8^⋆	2.3 × 10^−14^⋆	1.0
A1	1.3 × 10^−37^⋆	3.7 × 10^−2^‡	3.8 × 10^−33^⋆	1.7 × 10^−16^⋆
A2	2.4 × 10^−2^‡	1.8 × 10^−2^‡	3.9 × 10^−1^	2.8 × 10^−1^

^a^ Kruskal–Wallis test results.

^b^ Pairwise comparisons (PW) between hypotension and normotension.

^c^ PW between hypotension and hypertension.

^d^ PW between normotension and hypertension.

### Comparison between BP states using Kruskal–Wallis tests

Kruskal–Wallis tests, a nonparametric alternative to one-way ANOVA, were used to compare indices among BP states when measured using PRV or HRV. Pairwise Wilcoxon tests were used as post hoc analyses when the Kruskal–Wallis results indicated significant differences. The results are shown in Tables 4 and 5, and there were statistically significant differences among BP states from all indices, except for cHFx measured using HRV. The pairwise comparisons revealed that most of the indices showed differences among the three stages, especially when measured using PRV.

**Table 5:** Kruskal–Wallis and post hoc multiple comparisons *p* values for the comparison among blood pressure states from indices measured from heart rate variability. ‡: *p* value less than 5.00 × 10^−2^; †: *p* value less than 5.00 × 10^−3^; ⋆: *p* value less than 5.00 × 10^−4^.

Indices	KW^a^	Multiple comparisons		
		PW1 ^b^	PW2 ^c^	PW3 ^d^
AVNN	3.8 × 10^−61^⋆	3.5 × 10^−36^⋆	6.4 × 10^−1^	6.1 × 10^−60^⋆
SDNN	4.0 × 10^−53^⋆	4.8 × 10^−26^⋆	6.1 × 10^−51^⋆	3.2 × 10^−4^⋆
RMSSD	2.6 × 10^−63^⋆	4.0 × 10^−15^⋆	2.2 × 10^−63^⋆	1.3 × 10^−11^⋆
NN50	5.8 × 10^−46^⋆	5.3 × 10^−2^	2.9 × 10^−36^⋆	8.0 × 10^−21^⋆
pNN50	5.7 × 10^−46^⋆	5.1 × 10^−2^	2.5 × 10^−36^⋆	9.4 × 10^−21^⋆
VLF	4.2 × 10^−68^⋆	3.8 × 10^−19^⋆	3.1 × 10^−63^⋆	4.2 × 10^−16^⋆
LF	3.0 × 10^−64^⋆	2.2 × 10^−4^⋆	1.7 × 10^−57^⋆	1.6 × 10^−25^⋆
HF	3.8 × 10^−30^⋆	1.1 × 10^−1^	1.8 × 10^−26^⋆	1.2 × 10^−13^⋆
TP	5.6 × 10^−57^⋆	5.0 × 10^−9^⋆	1.3 × 10^−51^⋆	2.8 × 10^−19^⋆
nLF	7.1 × 10^−7^⋆	1.2 × 10^−6^⋆	2.4 × 10^−3^†	4.4 × 10^−3^†
nHF	1.4 × 10^−20^⋆	3.9 × 10^−9^⋆	3.1 × 10^−22^⋆	1.0
LF/HF	1.5 × 10^−17^⋆	3.3 × 10^−11^⋆	2.0 × 10^−16^⋆	2.7 × 10^−1^
cLF_x_	7.6 × 10^−17^⋆	6.9 × 10^−15^⋆	5.8 × 10^−11^⋆	7.9 × 10^−4^†
cHF_x_	5.5 × 10^−1^	-	-	-
cTP_x_	3.2 × 10^−17^⋆	2.4 × 10^−8^⋆	4.1 × 10^−18^⋆	1.0
cLF_y_	1.3 × 10^−65^⋆	8.3 × 10^−7^⋆	5.9 × 10^−61^⋆	2.5 × 10^−22^⋆
cHF_y_	8.9 × 10^−20^⋆	8.2 × 10^−8^⋆	1.1 × 10^−21^⋆	8.9 × 10^−1^
cTP_y_	2.0 × 10^−55^⋆	1.2 × 10^−46^⋆	2.9 × 10^−45^⋆	3.7 × 10^−1^
SpEn	9.8 × 10^−48^⋆	1.4 × 10^−48^⋆	3.9 × 10^−25^⋆	6.5 × 10^−10^⋆
S	5.7 × 10^−59^⋆	6.3 × 10^−2^	2.4 × 10^−50^⋆	2.4 × 10^−27^⋆
SD1	2.6 × 10^−63^⋆	4.0 × 10^−15^⋆	2.2 × 10^−63^⋆	1.3 × 10^−11^⋆
SD2	3.7 × 10^−61^⋆	3.8 × 10^−36^⋆	6.3 × 10^−1^	5.9 × 10^−60^⋆
SD1/SD2	3.1 × 10^−71^⋆	6.4 × 10^−61^⋆	2.1 × 10^−56^⋆	4.7 × 10^−1^
COM	2.7 × 10^−53^⋆	2.7 × 10^−26^⋆	4.6 × 10^−51^⋆	3.6 × 10^−4^⋆
BSE	8.6 × 10^−34^⋆	1.0	6.3 × 10^−24^⋆	1.0 × 10^−21^⋆
SSE	1.9 × 10^−23^⋆	9.7 × 10^−4^†	3.7 × 10^−10^⋆	1.9 × 10^−20^⋆
ApEn	4.2 × 10^−10^⋆	1.9 × 10^−10^⋆	7.1 × 10^−6^⋆	1.5 × 10^−2^‡
SampEn	4.7 × 10^−16^⋆	3.4 × 10^−17^⋆	1.7 × 10^−7^⋆	6.6 × 10^−5^⋆
MSE	1.5 × 10^−4^⋆	1.2 × 10^−3^†	1.0	2.3 × 10^−4^⋆
D2	1.2 × 10^−7^⋆	4.2 × 10^−2^‡	2.8 × 10^−3^†	5.5 × 10^−7^⋆
LYA	3.3 × 10^−24^⋆	3.3 × 10^−3^†	1.4 × 10^−22^⋆	7.8 × 10^−9^⋆
A1	4.8 × 10^−30^⋆	3.0 × 10^−6^⋆	7.0 × 10^−32^⋆	3.2 × 10^−5^⋆
A2	2.4 × 10^−2^‡	1.1 × 10^−1^	1.0	2.3 × 10^−2^‡

^a^ Kruskal–Wallis test results.

^b^ Pairwise comparisons (PW) between hypotension and normotension.

^c^ PW between hypotension and hypertension.

^d^ PW between normotension and hypertension.

**Table 6:** Ratio of agreement (BAR, %) derived from Bland–Altman analysis as suggested in refs. ^1,6^. Good agreement (*↑*): BAR < 10%; moderate agreeement ( ↔ ): 10% ≤ BAR < 20%; insufficient agreement (*↓*): BAR ≥ 20%.

Indices	Hypotension	Normotension	Hypertension
AVNN	3.92 × 10^−1^*↑*	5.01 × 10^−1^*↑*	4.43 × 10^−1^*↑*
SDNN	2.64 × 10^3^*↓*	1.81 × 10^3^*↓*	1.57 × 10^3^*↓*
RMSSD	5.12 × 10^3^*↓*	5.31 × 10^3^*↓*	4.29 × 10^3^*↓*
NN50	0.00*↑*	0.00*↑*	0.00*↑*
pNN50	0.00*↑*	0.00*↑*	0.00*↑*
VLF	2.27 × 10^−7^*↑*	5.01 × 10^−8^*↑*	7.81 × 10^−8^*↑*
LF	6.29 × 10^−7^*↑*	4.99 × 10^−7^*↑*	3.71 × 10^−7^*↑*
HF	1.36 × 10^−6^*↑*	1.33 × 10^−6^*↑*	9.33 × 10^−7^*↑*
TP	2.23 × 10^−7^*↑*	2.27 × 10^−7^*↑*	1.16 × 10^−7^*↑*
nLF	7.41 × 10^1^*↓*	6.44 × 10^1^*↓*	6.65 × 10^1^*↓*
nHF	4.47 × 10^1^*↓*	6.03 × 10^1^*↓*	4.53 × 10^1^*↓*
LF/HF	1.05 × 10^2^*↓*	1.37 × 10^2^*↓*	1.26 × 10^2^*↓*
cLF_x_	3.07 × 10^1^*↓*	3.96 × 10^1^*↓*	5.81 × 10^1^*↓*
cHF_x_	3.49*↑*	7.39*↑*	7.86*↑*
cTP_x_	2.14 × 10^2^*↓*	2.41 × 10^2^*↓*	2.40 × 10^2^*↓*
cLF_y_	6.08 × 10^−5^*↑*	4.00 × 10^−5^*↑*	2.63 × 10^−5^*↑*
cHF_y_	3.36 × 10^−4^*↑*	2.45 × 10^−4^*↑*	2.43 × 10^−4^*↑*
cTP_y_	1.70 × 10^−5^*↑*	8.54 × 10^−6^*↑*	2.83 × 10^−6^*↑*
SpEn	7.04 × 10^−2^*↑*	1.72 × 10^−1^*↑*	8.91 × 10^−2^*↑*
S	1.00 × 10^3^*↓*	1.01 × 10^3^*↓*	8.08 × 10^2^*↓*
SD1	7.24 × 10^3^*↓*	7.51 × 10^3^*↓*	6.07 × 10^3^*↓*
SD2	1.39 × 10^−1^*↑*	1.77 × 10^−1^*↑*	1.56 × 10^−1^*↑*
SD1/SD2	1.66 × 10^4^*↓*	1.61 × 10^4^*↓*	1.46 × 10^4^*↓*
COM	3.36 × 10^5^*↓*	2.27 × 10^5^*↓*	1.66 × 10^5^*↓*
BSE	1.58*↑*	1.41*↑*	1.37*↑*
SSE	2.90*↑*	3.12*↑*	3.49*↑*
ApEn	4.09 × 10^1^*↓*	9.03 × 10^1^*↓*	6.70 × 10^1^*↓*
SampEn	5.28 × 10^1^*↓*	1.39 × 10^2^*↓*	8.97 × 10^1^*↓*
MSE	3.80*↑*	5.14*↑*	2.71*↑*
D2	3.33 × 10^2^*↓*	1.17 × 10^3^*↓*	9.49 × 10^2^*↓*
LYA	4.15*↑*	4.58*↑*	1.85*↑*
A1	3.58 × 10^1^*↓*	3.17 × 10^1^*↓*	3.10 × 10^1^*↓*
A2	1.13 × 10^1^ ↔	9.12*↑*	7.47*↑*

### Bland–Altman analysis to assess agreement

**Fig. 2:**
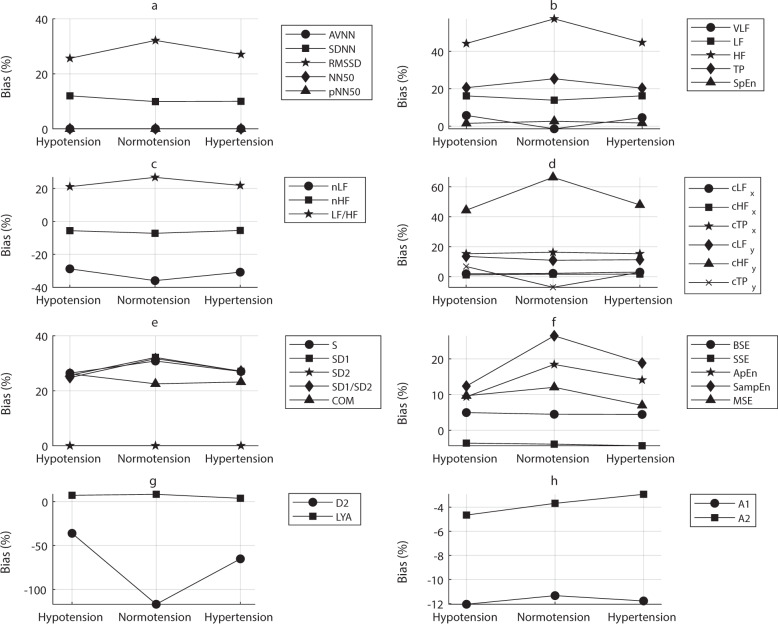
Bias measured from the Bland–Altman analysis comparing indices extracted from pulse rate variability and heart rate variability. The bias was obtained between measured indices when comparing pulse rate variability and heart rate variability in each blood pressure state (hypotension, normotension, and hypertension) using the Bland–Altman analysis. Indices analysed were **a** time-domain indices, **b** absolute and entropy indices from the frequency domain, **c** relative indices from the frequency domain, **d** centroid-related indices from the frequency domain, **e** Poincaré plot indices, **f** entropy-related indices, **g** phase-related indices, and **h** indices resulting from the detrended fluctuation analysis.

Because neither correlation analyses nor ANOVA could be used to evaluate the agreement between HRV and PRV measurements, Bland–Altman analyses, as proposed in^20^, were performed for each extracted index. Bias and LoAs were measured, and the results are summarized in Figs. 2 and3.

**Fig. 3:**
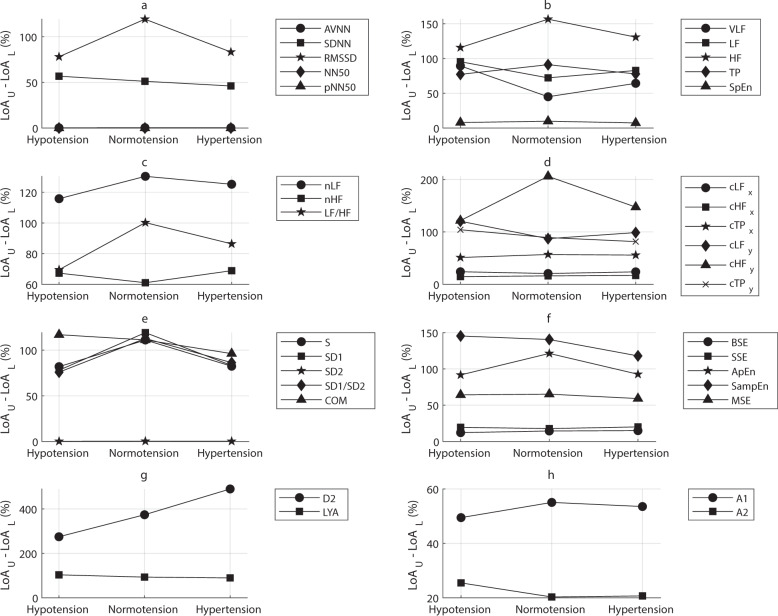
Differences between upper (LoA_U_) and lower (LoA_L_) limits of agreement obtained from Bland–Altman analysis comparing indices measured from pulse rate variability and heart rate variability. The limits of agreement were obtained from the Bland–Altman analysis performed with the extracted indices in each blood pressure state (hypotension, normotension, and hypertension). Indices analysed were **a** time-domain indices, **b** absolute and entropy indices from the frequency domain, **c** relative indices from the frequency domain, **d** centroid-related indices from the frequency domain, **e** Poincaré plot indices, **f** entropy-related indices, **g** phase-related indices, and **h** indices resulting from the detrended fluctuation analysis.

As shown in Fig. 2, most of the indices were overestimated when measured from PRV. Some others were underestimated, such as nLF, nHF, LF/HF, SSE, D2, A1, and A2. Indices associated with short-term changes were especially overestimated when measured from PRV. Although a general conclusion is difficult to be achieved, for most of the indices the bias differed according to the BP state. Interestingly, most indices showed a larger absolute bias during normotension. A similar trend was observed in the differences between the upper and lower LoAs, with large differences especially in indices associated with short-term changes. The largest differences were observed for D2, SampEn, and most of the frequency-domain indices. NN50 and pNN50 showed a bias and difference between LoAs of zero, which shows very good agreement between HRV and PRV.

The BAR results are shown in Table 6. For NN50 and pNN50, the bias and difference between LoAs were equal to zero for all conditions, and thus, the ratio was not measured because the agreement was total. The agreement tended to remain as good, moderate, or insufficient regardless of the BP state. Most of the indices that showed insufficient agreement are associated with short-term changes.

## Discussion

HRV has been proposed as a useful, noninvasive, indirect measurement of the cardiac ANS. It has been used for several decades as an indicator of parasympathetic and sympathetic activity^3^, and it has been studied as a biomarker for a broad range of diseases. However, it has been found that the measurement of HRV in real-life scenarios can be impaired by several conditions, especially because of the cumbersome instrumentation needed for the acquisition of the ECG signals, which has, to some extent, precluded the usefulness and acceptance of HRV as a tool for clinicians to diagnose and monitor diseases, and for larger public health applications^6^. Hence, several researchers have started to investigate the possibility of replacing HRV information with a very similar signal, PRV, which is based on pulse waves that are easier to obtain and more ubiquitous, such as PPG signals^9^. Nonetheless, the promise of PRV as a valid surrogate for HRV has been questioned, and some studies have concluded that, although they are very similar, PRV and HRV are not exactly the same and that PRV may not be a suitable surrogate for HRV, especially when measured in disease states and in older subjects^1,12^.

Various explanations for the differences between HRV and PRV have been given. Some authors argue that the differences are mainly due to processing issues, such as the identification of fiducial points from the PPG signal^21,22^, the sampling rate used for the acquisition of the signals^23–25^, and the processing techniques used for the analysis of PRV^26^. However, other authors have suggested that, although these factors may affect PRV, physiological issues may have a more profound effect on the differences between these two signals^12,27^. The relationship between HRV and PRV may be affected by not only PTT but also other factors, such as external forces on the arterial vessels^28^, the presence of pathologies, including cardiovascular disorders^28–30^, and the body location at which PRV is being measured^8,31^. An important contributor to these differences is respiratory activity, which affects vasoconstriction and modulates aortic and left-ventricular pressure, altering the time of opening of the aortic valve during the cardiac cycle^32^. Nonetheless, several processes take part in the information transmission from the pure electrical ECG and the R waves to the mechanical PPG pulse wave, as is explained in ref. ^33^. These factors may also explain in part the differences observed between HRV and PRV, especially under non-resting conditions and in non-healthy, older subjects. Hence, PRV should not be considered a surrogate of HRV, but should be treated as an independent biomarker instead, which may contain additional information not available in HRV^33^.

Thus, because of the differences observed in previous studies between HRV and PRV, the aim of this study was to assess the relationship between these two signals in critically ill subjects hospitalized in intensive care units. Because these subjects exhibited changes in BP, PRV, and HRV were also compared when the subjects experienced normotension or hyper- and hypotensive events.

A first analysis was performed comparing PRV and HRV indices using linear correlation. Among the time-domain indices, an almost perfect correlation was observed between HRV and PRV for AVNN, regardless of BP state. For SDNN, NN50, and pNN50, higher correlations were observed as BP increased, but pNN50 had the lowest correlation of the time-domain indices in all BP states. RMSSD, which reflects short-term changes in HRV and PRV^2^, had a lower correlation, with a correlation coefficient below 0.8, during normotension.

The frequency-domain indices exhibited different trends. Absolute indices (VLF, LF, HF, and TP) and the ratio between LF and HF (LF/HF) had stable and high correlations. For normalized indices, on the other hand, there were differences according to the BP state, with better correlations during normotension. Spectral entropy (SpEn) exhibited a behavior similar to that observed for RMSSD. Indices related to the centroid of the frequency bands in the y-coordinate were relatively stable, while the x-coordinate of the centroids featured the worst correlations, especially for the x-coordinate of the HF band centroid.

The correlations for the nonlinear indices were, in general, worse than those for the time- and frequency-domain indices. This was especially true for the phase-related indices, the correlation dimension and the Lyapunov exponent. Among the Poincaré plot indices, SD2 had a nearly perfect correlation between HRV and PRV, while SD1/SD2 and SD1 exhibited behaviors similar to that of RMSSD. The correlations for the entropy-related indices were also relatively poor. The correlation for the BSE values was strongly affected by normotension, and although SampEn, ApEn, and SSE were stable regardless of the BP state, their correlation coefficients were low. Finally, A1 and A2 from the detrended fluctuation analysis behaved similarly to SD1 and SD2, respectively, probably because of the differences between the short- and long-term changes in HRV and PRV.

PRV and HRV were also compared using a Friedman rank sum test, as a nonparametric alternative of a repeated-measured ANOVA. The results indicated that there were differences between PRV and HRV in all BP states for all indices, except for the measurements of VLF and cTPy, which showed nonsignificant differences between HRV and PRV during normotension. These two indices need to be considered with care because they are probably a reflection of long-term changes, especially VLF, and require recordings longer than 5 min. In general, these results indicate that PRV and HRV are not the same, regardless of the BP state.

In addition, it was also determined if there were individual differences in PRV and HRV among BP states. The Kruskal–Wallis test results revealed that, in general, both HRV and PRV were different among hypotension, normotension, and hypertension states. The only index for which there was not a statistically significant difference was cHFx when measured from HRV. Based on the post hoc comparisons, it was concluded that PRV showed more differences than HRV. Again, as was observed for the correlations, these differences were especially observed in the nonlinear indices and in indices reflecting short-term changes, such as RMSSD, SD1, SpEn, and A1. Interestingly, most of the differences were observed when normotension was compared to either of the two other BP states.

Finally, agreement between HRV and PRV was assessed using Bland–Altman analysis and three measurements were obtained from these to evaluate the agreement between HRV and PRV: the bias, difference between limits of agreement, and BAR. For the time-domain indices, SDNN and RMSSD were overestimated when using PRV, whereas AVNN, NN50, and pNN50 had a bias close to zero. The absolute-power frequency-domain indices were also overestimated when using PRV, especially HF, LF/HF, and TP. On the contrary, the relative power indices were usually underestimated. The y-coordinate of the centroid of the HF band was also largely overestimated when using PRV. This same trend was observed for all Poincaré-plot indices, as well as for SampEn and ApEn, whereas SSE, D2, and both A1 and A2 were underestimated. The degrees of over- and underestimation tended to be larger during normotension. This same trend was observed for the limits of agreement: larger differences were observed during normotension and when some short-term indices, such as RMSSD, HF, cHFy, SD1, and A1, were measured.

The obtained BARs indicated good agreement for AVNN, VLF, LF, HF, TP cHFx, cLFy, cHFy, cTPy, SpEn, SD2, BSE, SSE, MSE, LYA, and A2. Insufficient agreement was observed for SDNN, RMSSD, nLF, nHF, LF/HF, cLFx, cTPx, S, SD1, SD1/SD2, COM, ApEn, SampEn, D2, and A1. Most short-term indices showed an extremely large BAR, which indicates a very poor agreement between HRV and PRV for the measurement of these indices in critically ill patients regardless of the BP state. There was no indication that BP changes caused significant changes in the agreement.

In conclusion, the obtained results indicate that PRV and HRV were not the same regardless of the BP state of the subjects, especially when nonlinear indices and indices associated with short-term changes were analyzed, which agrees with the results of previous studies^8,14,30,34^. Interestingly, the differences tended to be larger during normotension. However, although they are not the same and PRV tends to over- or underestimate HRV, both signals behave similarly in most cases. Nonetheless, the Kruskal–Wallis results indicate that PRV seems to be more sensitive to changes in BP. This could be considered as an indication that PRV contains additional information not available in HRV, which might help increase the applicability of the technique in clinical scenarios. Moreover, the widespread use of PPG in wearable devices is generating a lot of research with PRV, which is aiming to apply this more practical technique, in comparison with HRV, for the diagnosis and monitoring of several physiological phenomena related to disease (i.e., cardiovascular disease, mental health). This, in turn, shows the applicability and potential of PRV for public health studies and, hence, for screening subjects that may need later further analyses in the clinical setting, with more specialized tools.

Future studies are needed to clarify the origin of the differences between HRV and PRV and to evaluate the capability of PRV to identify BP states, which aid in the noninvasive, continuous measurement of BP. Moreover, it is critical that measurement and analysis guidelines and standards are adopted for PRV studies. This would enhance the quality of the research in this field, allowing the comparability among results obtained in different studies, and possibly increasing the applicability of PRV in clinical settings.

This study has several limitations. First, the signals used were obtained from an available database from Physionet. Thus, several variables were not controlled for, and although all subjects were hospitalized in an intensive care unit, their diagnosis was unknown, which may have affected the results. Another limitation involves the segmentation of the data into 5-min-long segments, which may have been too short for the extraction of some indices, especially frequency-domain indices. However, this length was considered necessary to obtain as many segments as possible during each BP state and still ensure sufficient data for the PRV and HRV analysis. Moreover, an overlap of 10 s was used to separate the segments, which might have been too short to reflect BP changes. Again, this was done to produce a larger database. Another limitation of the study involves the classification of segments in each BP state, specifically determining exactly which state was predominant in each segment, especially in subjects who exhibited two or more BP states during the entire recording. A larger number of available segments might have helped to mitigate this effect, and outliers for each PRV and HRV index were corrected. It is important to mention that MIMIC-III database lacks synchronicity^35^, which could bias the results obtained in this study. Finally, it is also worth noting that some of the extracted indices were not optimized, especially the nonlinear indices such as Poincaré-plot indices, BSE, SSE, phase indices, and DFA-related indices. Using an optimization procedure for these indices might lead to different results than those obtained in this study, as can be seen in ref. ^30^.

## Methods

### Signal selection

A subset of 500 records was obtained from the MIMIC-III Waveform Database^18,19^. Each record in the subset contained the ECG, PPG, and invasive arterial BP (ABP) signals, which were obtained at a 125 Hz sampling rate, from critically ill subjects in adult intensive care units. As the MIMIC-III is a publicly available database, ethical approval was not required for this study.

**Fig. 4:**
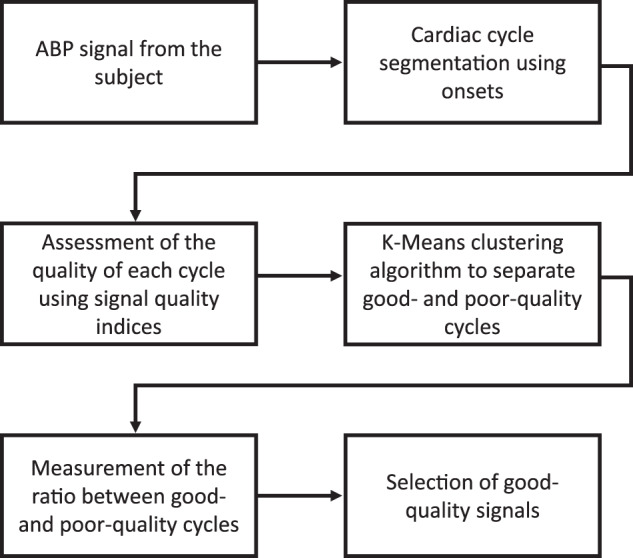
Signal quality assessment algorithm. This algorithm was applied for discarding low-quality arterial blood pressure signals.

These records were filtered to reject PQ signals and signals with length of less than 5 min. First, signals with a duration of less than 5 min were discarded. Then, a signal quality index (SQI) algorithm was employed to detect good- and poor-quality ABP signals, as shown in Fig. 4. In the signal quality assessment algorithm, the onsets from each ABP signal were detected by applying the algorithm described in ref. ^36^, and the cardiac cycles were obtained. Then, the quality of each cardiac cycle was assessed using SQIs proposed in the literature^37–39^. A K-means clustering algorithm was employed to automatically group good-quality (GQ) and poor-quality (PQ) cardiac cycles in two clusters, with the SQIs used as features. Because it was expected that most of the cycles would be of good quality, the larger cluster was considered the GQ cluster. Then, the ratio (*R*_GQ_) between the number of cycles grouped as “good-quality cycles” and the total number of cycles was obtained as in (Eq. 1). The records with a *R*_GQ_ greater than or equal to 80% were considered GQ signals, and the remaining records were discarded.

1 $${R}_{{\mathrm{GQ}}}=(100 \% )\frac{{n}_{{\mathrm{GQ}}}}{{n}_{{\mathrm{GQ}}}+{n}_{{\mathrm{PQ}}}}$$

### Signal processing

MATLAB^®^ (version 2020a) was used for signal processing. ECG, PPG, and ABP signals were segmented into 5-min-long segments, with an overlap of 10 s between consecutive segments.

**Fig. 5:**
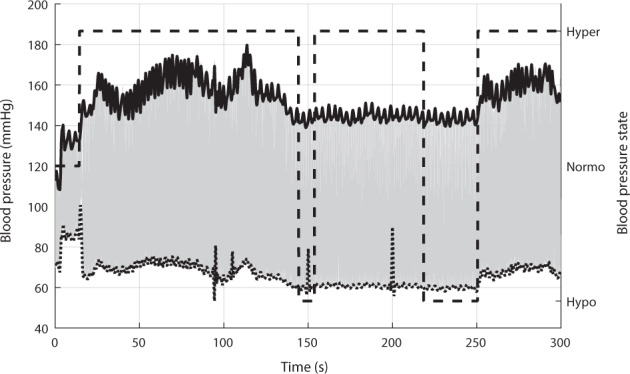
Arterial blood pressure signal analysis. Example of the analysis of a 5-min arterial blood pressure (ABP, gray line) signal, with the trends for systolic (SBP, continuous line) and diastolic (DBP, dotted line) blood pressure, as well as the determination of blood pressure state (BP state, dashed line). Hypo hypotension, Normo normotension, Hyper hypertension.

After segmentation, 5-min-long ABP signals were filtered using a 12 Hz, fourth-order, lowpass Butterworth filter. Peaks and onsets were detected, corrected, and interpolated using a cubic spline to obtain systolic blood pressure (SBP) and diastolic blood pressure (DBP) trends . From the SBP and DBP information, events of hypertension (SBP greater than 140 mmHg or DBP greater than 90 mmHg) and hypotension (SBP lower than 90 mmHg or DBP lower than 60 mmHg) were identified. Then, each 5 min segment was labeled as hypertension, normotension, or hypotension according to the most frequent label in each 5 min segment. An example of these trends and labels is shown in Fig. 5.

**Fig. 6:**
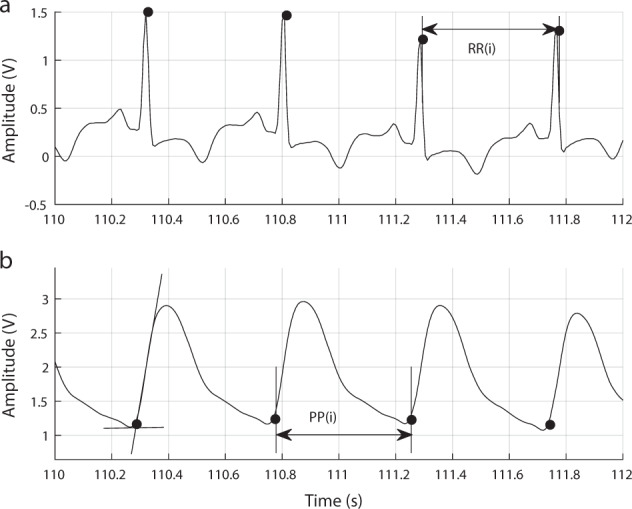
Electrocardiography and photoplethymography analysis for the extraction of heart rate variability and pulse rate variability, respectively. Example of **a** an electrocardiography (ECG) and **b** a photoplethysmography (PPG) signal. R peaks (black circles on the ECG signal) were detected from ECG signals to measure heart rate variability (HRV) as the time interval between consecutive R peaks (RR intervals). Onsets (black circles on the PPG signal) were detected from PPG signals to measure pulse rate variability (PRV) as the time interval between consecutive onsets (PP intervals).

For HRV analysis, R peaks were detected from each 5-min segment obtained from the ECG signals, using the algorithm proposed in ref. ^40^. HRV was measured as the time difference, in milliseconds, between consecutive R peaks. For the frequency-domain analysis, the uneven HRV series was interpolated using a cubic spline interpolation and a sampling rate of 4 Hz, and the power spectrum was obtained using the Fast Fourier Transform (FFT). Outliers in both the original and interpolated time series were defined as values higher or lower than the mean value plus or minus 1.96 times the standard deviation of the series. These outliers were then replaced with the mean value of the five previous values in the time series. Figure 6a illustrates this process.

Similarly, after segmentation, the onset of each cardiac cycle from the 5-min-long PPG signals was obtained as the intersection point of the tangent lines arising from the maximum slope point and the valley of the waveform. This fiducial point was selected because of its robustness for PRV analysis^21,41^. PRV was measured as the time difference, in milliseconds, between consecutive onsets from the PPG signal, as shown in Fig. 6b. Similar to the method used for HRV trends, PRV was interpolated using a 4 Hz sampling rate and a cubic spline interpolation, and outliers were detected and corrected. Again, FFT was used to obtain the power spectrum.

As was explained previously, time- and frequency-domain indices and nonlinear indices extracted from Poincaré plot, entropy, phase, and detrended-fluctuation analyses were obtained, as summarized in Table 1.

### Statistical analysis

All statistical analyses were performed in MATLAB^®^ and R (version 3.6.1). A significance level of 5% (*p* value < 0.05) was considered significant for all analyses, and the normality of data was assessed using a Lilliefors test.

The aim of this study was to assess the differences between HRV and PRV indices extracted from critically ill subjects during hypo-, normo-, and hypertensive events. Hence, the level of the linear relationship between PRV and HRV indices was evaluated using the Spearman correlation coefficient. The differences between HRV and PRV were also evaluated using Friedman rank sum tests, and the differences among BP states were assessed using Kruskal–Wallis tests, with pairwise Wilcoxon tests with Bonferroni correction as post hoc analyses.

Moreover, because a good correlation does not imply good agreement, the agreement between HRV and PRV during each of the BP states was assessed using Bland–Altman analysis^20^. From the Bland–Altman plots, the bias and difference between limits of agreement (LoAs) were obtained, and the ratio of agreement (BAR) was measured using (Eq. 2) and (Eq. 3), as suggested in refs. ^8,42^. Agreements were categorized as good (BAR ≤ 10%), moderate (10% < BAR ≤ 20%), or insufficient (BAR > 20%).

2 $${\mathrm{LoA}}=\overline{x}\pm 1.96{\sigma }_{x},x={\rm{HRV}}-{\rm{PRV}}$$

3 $${\mathrm{BAR}}=(100 \% )| \frac{1.96{\sigma }_{x}}{\overline{{\rm{HRV}}+{\rm{PRV}}}}| ,x={\rm{HRV}}-{\rm{PRV}}$$

### Reporting Summary

Further information on research design is available in the Nature Research Reporting Summary linked to this article.

## Supplementary information

Reporting Summary

## Data Availability

The data that support the findings of this study are available in PhysioNet, https://archive.physionet.org/physiobank/database/mimic2wdb/.

## Code availability

The custom code used in this study can be requested contacting the corresponding author via e-mail.
